# Exploring two tumor treatment strategies: effectiveness of ribosome inactivating proteins and mesenchymal stem cells/MSC derived extracellular vesicles in cancer treatment

**DOI:** 10.3389/fonc.2025.1533065

**Published:** 2025-05-13

**Authors:** Maryamosadat Mavaei, Simin Farokhi, Mohammad Hasan Yousefi, Arshia Fakouri, Alireza Shadab, Mohammad Hossein Abdolmohammadi, Faranak Fallahian, Hamed Afkhami

**Affiliations:** ^1^ Student Research Committee, Kermanshah University of Medical Sciences, Kermanshah, Iran; ^2^ Pharmaceutical Sciences Research Center, Health Institute, Kermanshah University of Medical Sciences, Kermanshah, Iran; ^3^ Student Research Committee, USERN Office, Lorestan University of Medical Sciences, Khorramabad, Iran; ^4^ Student Research Committee, Qom University of Medical Sciences, Qom, Iran; ^5^ Department of Tissue Engineering and Applied Cell Sciences, School of Medicine, Qom University of Medical Sciences, Qom, Iran; ^6^ Cellular and Molecular Research Center, Qom University of Medical Sciences, Qom, Iran; ^7^ Department of Immunology, School of Medicine, Iran University of Medical Sciences, Tehran, Iran; ^8^ Iran University of Medical Sciences, Deputy of Health, Tehran, Iran; ^9^ Nervous System Stem Cells Research Center, Semnan University of Medical Sciences, Semnan, Iran; ^10^ Department of Medical Microbiology, Faculty of Medicine, Shahed University, Tehran, Iran

**Keywords:** ribosome-inactivating proteins (RIP), mesenchymal stem Cell (MSC), MSC-derived extracellular vesicles (EVs), cancer, cancer treatment

## Abstract

Cancer is a complex and heterogeneous disease that often requires multifaceted treatment strategies to achieve optimal therapeutic outcomes. Given the limitations of single-agent therapies, particularly in the face of intricate biological signaling networks and treatment resistance, there is a growing need for combinatory approaches. This article presents a novel hypothesis: the simultaneous use of ribosome-inactivating proteins (RIPs) and mesenchymal stem cells (MSCs) or MSC-derived extracellular vesicles (EVs) in cancer treatment. RIPs, with their potent cytotoxic properties, can target tumor cells effectively, while MSCs, known for their tumor-homing abilities and regenerative potential, can serve as delivery vehicles, potentially enhancing the targeting precision and reducing the systemic toxicity of RIPs. This hypothesis explores the synergistic potential of combining these two therapeutic modalities, leveraging the advantages of both techniques to create a more effective cancer treatment strategy. By combining RIPs’ ability to inhibit protein synthesis with MSCs or MSC-derived EVs’ capability to modulate the tumor microenvironment and deliver therapeutic agents. This approach offers a promising avenue for overcoming cancer’s inherent complexity. However, challenges remain, such as optimizing dosing protocols, addressing safety concerns, and ensuring efficient drug delivery. Future research and clinical trials are necessary to validate this combination as a viable cancer therapy.

## Graphical Abstract

The effect of RIP and MSC on cancer

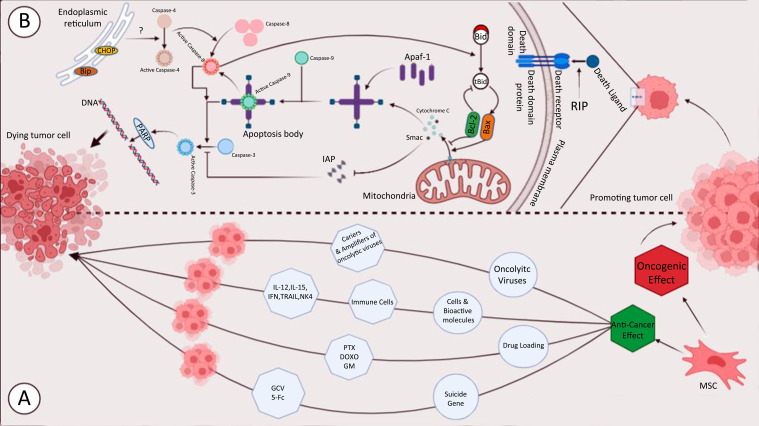

## 1 Introduction

Cancer has emerged as a significant public health issue, with more than 52,900 new diagnoses and over 27,000 deaths each day. Projections suggest that by 2040, the number of new cancer cases will reach 28 million annually, with 16.2 million deaths globally. To effectively decrease cancer mortality rates worldwide, it is essential to focus on the broad adoption of personalized and targeted treatments, coupled with increased investment in the development of advanced cancer therapies (1). These alarming statistics surrounding cancer have intensified efforts to either prevent the disease or detect it at an early stage, when treatments tend to be less invasive, more costeffective, and have higher chances of success. However, the precise mechanisms by which tissues undergo malignant transformation remain a topic of debate and controversy, complicating efforts toward cancer prevention and early intervention (2, 3). Cancer, a serious and often life-threatening condition, is marked by the uncontrolled proliferation and spread of abnormal cells within the body. The disease poses major challenges for both patients and their families, impacting them not only physically but also emotionally and financially (4–6). The growing incidence of cancer has also increased the economic burden on healthcare systems worldwide. Projections indicate that global cancer drug sales will soar from $193 billion USD in 2022 to $377 billion USD by 2027. This increase is driven by earlier cancer detection, improved survival rates that extend treatment durations, broader access to innovative cancer therapies, and the ongoing introduction of new drugs into the market (7). Conventional cancer treatments face significant limitations due to their associated radiotoxicity (such as xerostomia, hepatotoxicity, or pneumonia) and adverse effects, including genetic mutations, cytotoxicity, and the development of drug resistance (8). Advanced anticancer therapies require the development of targeted biological strategies in order to maximize efficacy while reducing toxicity (9).

While various cancer therapies have shown success in clinical applications, significant challenges such as drug resistance and low response rates remain unresolved. To address these issues, ongoing research focuses on discovering new therapeutic agents. Among these, ribosomeinactivating proteins (RIPs) have gained remarkable attention due to their potent anticancer properties. RIPs can inhibit protein synthesis and induce tumor cell death at extremely low concentrations, making them promising candidates for cancer treatment, provided they can effectively target cytosolic ribosomes (10–12). Recent developments in genetic recombination technology have facilitated the large-scale production of recombinant RIPs, simplifying the creation of various fusion proteins. Building on these technological advances, RIPs with enhanced druggability have been designed. Despite these improvements, the clinical application of RIPs for cancer treatment continues to face major hurdles. The main challenge is effective drug delivery, hindered by biological barriers within the body, including vascular, intratumoral, and intracellular barriers. To overcome these obstacles, innovative drug delivery systems (DDS) based on prodrug strategies have been developed (10).

Mesenchymal stem cells (MSCs) have become a promising tool for delivering therapeutic agents directly to tumor sites due to their unique properties, making them an effective approach for drug delivery strategies. They exhibit unique biological properties, such as differentiation capabilities, easy isolation, large-scale expansion, immune system modulation, and tumortargeting abilities. However, conflicting reports on their tumor-suppressive and -supportive effects pose challenges for their clinical application in cancer therapy (13–15).

Recent studies have indicated that MSCs may also exhibit additional beneficial effects through paracrine pathways, involving the generation and release of soluble factors and extracellular vesicles (EVs) mediating immune-modulatory and trophic functions (16–18). Emerging research highlights the potential of exosomes derived from MSCs as a promising cell-free alternative to traditional MSC-based therapies. These exosomes have garnered attention for their ability to mediate the therapeutic effects of MSCs while offering distinct advantages, including improved safety profiles, ease of storage, simplified transportation, and more convenient administration compared to MSC transplantation methods. As nanoscale vesicles, MSC-derived exosomes can encapsulate and deliver diverse biomolecules such as nanomedicines, functional proteins, mRNAs, and microRNAs (miRNAs) (14, 19, 20).

Cancer, with its diverse nature, rapid development of treatment resistance, and complex cellular pathways, poses significant challenges for single-agent therapies. It has become increasingly clear that the future of effective cancer treatment lies in combinatorial therapies (7, 21, 22). In the present scenario, the prime focus is on exploring potential of RIPs and MSCs/MSC-EVs to treat cancer. It reviews strategies aimed at minimizing the systemic toxicity of RIPs and addresses the current limitations of MSC therapy, while also highlighting the promise of combinatorial approaches. The review emphasizes how RIPs and MSCs/MSC-EVs can complement each other through synergistic mechanisms to offer a more effective therapeutic strategy. However, it is crucial to acknowledge that, while these approaches show considerable promise in preclinical studies, their success in clinical settings may vary.

## 2 Components of the tumor microenvironment

Cancers are complex ecosystems composed of tumor cells along with a variety of noncancerous cells, all embedded within a modified extracellular matrix (23). In addition to the tumors itself, it’s essential to study the dynamic tumors microenvironment (TME). The TME is primarily made up of malignant cells, endothelial cells, immune cells, diverse stromal cells, and extracellular matrix (ECM) components. Paget introduced the “seed and soil” hypothesis a decade ago, likening tumor cells to seeds and the tumor microenvironment to soil, underscoring the strong interdependence between tumor cell growth and their surrounding environment (24). Once thought to be mere bystanders in tumorigenesis, these host cells are now recognized as key contributors to cancer pathogenesis. The cellular makeup and functional state of the TME can vary widely based on the organ of origin, the inherent properties of the cancer cells, tumor stage, and patient-specific factors (23). Consequently, the TME is crucial in influencing tumor development, invasion, and the effectiveness of anti-cancer therapies (24). Before delving into the therapeutic strategies discussed in this review, we will first provide an overview of the essential components of the TME. Targeted anticancer agents and chemotherapies exploit inherent vulnerabilities in cancer cells. However, evidence suggests that the local TME can influence tumor behavior, either promoting or inhibiting its growth. Drugs that target cancer cell weaknesses have also been shown to affect the TME. Understanding these effects can help develop drug combinations that not only directly inhibit cancer cells but also exert indirect anticancer effects on the TME (25).

Recent studies have revealed that the TME’s cellular interactions play a role in cancer’s start and development. Malignant TME cells fail to effectively stimulate or target the immune system during the early stages of tumor development. Over time, these cells acquire the ability to evade the natural immune response and start inhibiting the adaptive immune response (26–28).

## 3 RIPs in cancer therapy

RIPs are a family of cytotoxic enzymes known for their ability to inhibit protein synthesis in eukaryotic cells. They achieve this by cleaving a specific adenine residue from the 28S rRNA within the 60S ribosomal subunit through N-glycosidase activity, thereby blocking the function of the ribosome. Initially discovered in the castor oil plant, Ricinus communis, RIPs have since been identified in a wide range of higher plants, and to a lesser extent, in fungi and bacteria (29). RIPs have been identified across a broad spectrum of plant species, spanning approximately 17 families, as well as in bacteria, fungi, and algae. Moreover, certain animal tissues have also been reported to exhibit RIP-like activity. A significant concentration of RIPs has been discovered within specific plant families, such as Caryophyllaceae, Sambucaceae, Euphorbiaceae, Cucurbitaceae, Poaceae, Phytolaccaceae, and Rosaceae. RIPs have also been localized in various plant tissues, including leaves, seeds, roots, and tubers. Bacterial species are known to produce type II RIPs, such as Shiga toxins. For example, specific strains of *Escherichia coli* synthesize Shiga toxin type 1 (Stx1) and Shiga toxin type 2 (Stx2), exhibiting enzymatic activity analogous to their plant counterparts. Similarly, several fungal species, including *Volvariella volvacea*, *Flammulina velutipes*, *Hypsizigus marmoreus*, and *Lyophyllum shimeji*, have been shown to produce RIPs.

Additionally, a RIP has been identified in algae, specifically *Laminaria japonica A*. Notably, RIPrelated genes have also been detected in the genomes of two mosquito species (30).

Various techniques have been employed for the purification of RIPs, tailored to specific sources and applications. Tobacco RIP (TRIP), isolated from *Nicotiana tabacum* leaves, was purified using ion exchange and gel filtration chromatography in combination with yeast ribosome depurination assays (31). Moschatin, a novel RIP from pumpkin *Cucurbita moschata* seeds, was purified through a series of steps, including ammonium sulfate precipitation, CM-cellulose 52 column chromatography, Blue Sepharose CL-6B affinity chromatography, and FPLC sizeexclusion chromatography (32). Similarly, an antiviral 25 kDa RIP from *Celosia plumosa* leaves were purified using a combination of 60% ammonium sulfate precipitation, FPLC-based anion and cation exchange chromatography with 10 and 50 mM NaCl, size-exclusion chromatography in 50 mM NaCl, and SDS-PAGE (10%) (33). MAP30, a type-I RIP from *Momordica charantia* was cloned, expressed in *Escherichia coli*, and purified to over 95% purity using Ni–NTA affinity chromatography (34).

RIPs are classified into three types based on their structure, mature proteins, isoelectric point (pI) value, molecular weight, and function. Type I RIPs are single-chain proteins, approximately 30 kDa in size, that possess N-glycosidase activity, such as trichosanthin (TCS) and cucurmosin. TCS, also known as monorcharin, is an example of a type 1 RIP with catalytic activity due to its solitary polypeptide chain. Type II RIPs, like ricin and abrin, are more complex, consisting of an enzymatic A-chain similar to Type I RIPs, linked to a B-chain with lectin properties that target specific sugar molecules. Type III RIPs, like JIP60 and maize ribosome-inactivating protein, a less common group, have been identified only in maize and barley, and their additional domains are not yet fully understood (35–37).

In recent years, RIPs have attracted significant attention for their potential use in cancer therapy. Some RIPs have demonstrated strong cytotoxicity towards cancer cells while exhibiting lower toxicity towards normal cells. They primarily induce apoptosis in cancer cells, though the precise mechanisms are still under investigation (36).

The toxic effects of certain type 2 RIPs, such as the toxicity of castor and jequirity beans to humans and animals, as well as the abortifacient properties of some *Cucurbitaceae* species, have been recognized since ancient times, long before the underlying proteins were identified. Until the early 1970s, the primary focus of scientific interest in plant RIPs was their toxicity. Subsequently, numerous RIPs were isolated and classified based on their structural and toxicological characteristics, with type 2 RIPs often demonstrating greater toxicity compared to type 1 RIPs. Notably, the cellular toxicity of type 2 RIPs generally aligns with their toxicity to animals and humans, albeit with some exceptions (38).

Type 2 RIPs are composed of two functional components: a toxic A-chain (N-β-glycosylase) and a cell-binding B-chain, which are connected by an interchain disulfide bond and hydrophobic interactions. The B-chain, which possesses lectin activity, binds to galactose residues on the surface of mammalian cells, facilitating the entry of the A-chain into the cells to exert its cytotoxic effects. In contrast, type 1 RIPs lack the lectin-containing B-chain, which significantly limits their ability to penetrate cells and results in lower cytotoxicity. Additionally, noncanonical type 3 RIPs have been identified in members of the *Poaceae* family. Examples include barley JIP-60, which contains a C-terminal domain of unknown function, and maize b-32, which requires proteolytic activation to achieve enzymatic functionality (39).

Despite their potential as innovative anti-tumor agents, RIPs also exhibit serious toxic side effects, including systemic anaphylaxis, immunogenicity, and general toxicity (29). A major challenge in developing RIPs for therapeutic use is their nonspecific toxicity, which poses a significant risk to healthy cells during treatment. Furthermore, RIPs require effective cellular uptake to exert their toxic effects, but they often lack the ability to independently cross the plasma membrane. This limitation highlights the need for advanced delivery systems capable of targeting specific cells while sparing healthy tissues. The upcoming sections will explore the mechanism of action, discuss the challenges posed by various barriers, and propose potential solutions to address these issues.

### 3.1 RIPs mechanism of action

RIPs with RNA N-glycosidase activity have attracted significant attention in cancer research. Since the identification of RIPs as potent toxins derived from Ricinus communis in the 1990s, numerous RIPs have been isolated and characterized from various plant and fungal species. These proteins, including ricin, sarcin, and trichosanthin, primarily function through depurination. RIPs target ribosomal subunit binding domains, where they cleave the sarcin-ricin loop in rRNA, a region essential for ribosome assembly and GTP hydrolysis. By impairing GTP hydrolysis, RIPs disrupt the energy supply required for the ribosome’s unidirectional movement during the elongation stage, thereby inhibiting elongation factors, halting protein synthesis, and ultimately inducing cell death (40).

RIPs play a critical role in inhibiting protein synthesis by targeting the eukaryotic ribosome, a process that requires their entry into the cell. The entry mechanism of RIPs varies depending on the type. Type II RIPs are known to bind to glycoproteins and glycolipids on the cell membrane, facilitating their entry via endocytosis. Once inside, they are transported from the Golgi apparatus to the endoplasmic reticulum through retrograde transport (41). The enzymatic components of these RIPs are released into the cytosol after exploiting the ER-associated degradation pathway, allowing them to reach the ribosomes and exert their inhibitory function (35).

In contrast, Type I RIPs, which lack sugar-binding activity, face more challenges in entering cells. They may enter to some extent by interacting with phospholipids in the cell membrane, but this process is not well understood. To enhance their entry, Type I RIPs are often conjugated with carriers like monoclonal antibodies, enabling them to specifically target and kill cells, particularly in cancer therapies. This has led to the development and study of various immunotoxins that leverage Type I RIPs for targeted cancer treatment. Once inside the cell, RIPs can induce apoptosis through multiple signaling pathways. The primary pathways include mitochondrial-mediated apoptosis, death receptor-mediated apoptosis, and ER stress-mediated apoptosis (29).

### 3.2 Cellular Mechanism of RIPs

#### 3.2.1 Mitochondrial-mediated apoptosis

This pathway involves the generation of reactive oxygen species (ROS) and the disruption of intracellular Ca^2+^ balance, leading to changes in mitochondrial membrane potential and permeability. These changes trigger the release of proapoptotic factors such as cytochrome c, apoptosis-inducing factor (AIF), and second mitochondriaderived activator of caspases (Smac), which activate caspase-9 and downstream caspase-3, leading to DNA fragmentation and cell death (29, 42).

#### 3.2.2 Death receptor-mediated apoptosis

This pathway is initiated when death receptors on the cell surface, such as Fas, bind to their respective ligands. This binding activates procaspase-8, which then activates caspase-3, driving the cell into apoptosis. In certain cells, RIPs can trigger this pathway, amplifying the apoptotic signal and leading to mitochondrial collapse (43).

#### 3.2.3 ER stress-mediated apoptosis

When cells are exposed to stressors such as toxins or hypoxia, misfolded proteins accumulate in the ER, leading to ER stress. RIPs can exacerbate this stress, resulting in the activation of ER stress-related proteins like Bip and CHOP, which subsequently activate caspase-4 and caspase-12. This pathway is another route through which RIPs can induce apoptosis, particularly in cancer cells (44).

**Figure 1:**
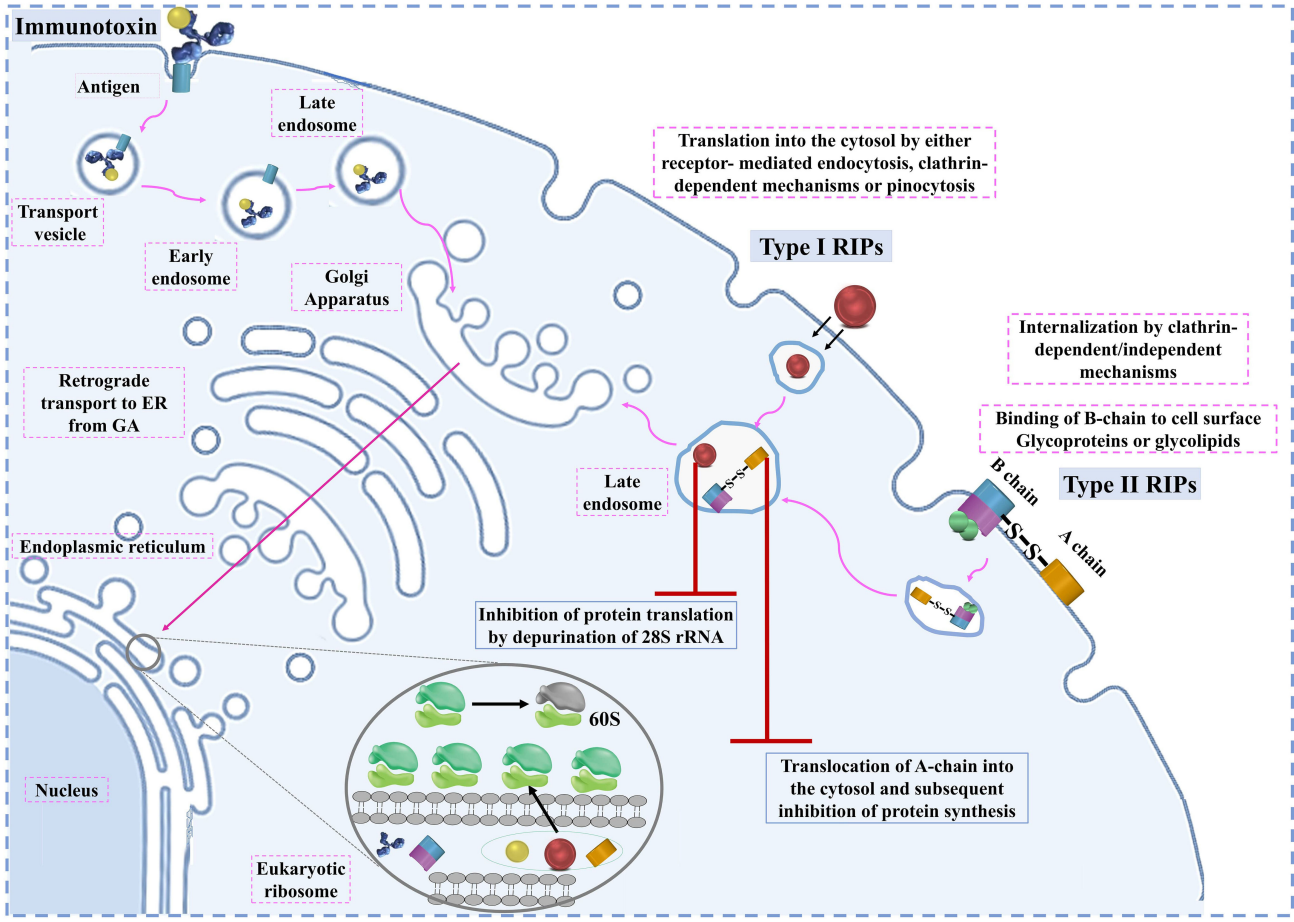
The entry pathways of type I RIPs, type II RIPs, and immunotoxins.

Overall, RIPs utilize these cellular mechanisms to induce apoptosis in target cells, making them potent candidates for cancer therapy. However, understanding these mechanisms in greater detail is essential for improving the effectiveness and specificity of RIP-based treatments in clinical settings. The entry pathways of type I RIPs, type II RIPs, and immunotoxins are shown in **Figure 1**. cellular mechanisms of RIPs are presented in **Table 1**.

**Table 1:** Cellular mechanisms of RIPs in inducing apoptosis.

Mechanism/Pathway	Description	Advantages	Disadvantages	Ref.
Entry Mechanism	**Type I RIPs:** Limited entry due to lack of sugar-binding activity; entry possible via phospholipid interaction or through conjugation with carriers like monoclonal antibodies. **Type II RIPs:** Enter cells via endocytosis, bind to glycoproteins/glycolipids, and use retrograde transport from Golgi to ER.	**Type I RIPs:** Conjugation with carriers can target specific cells, enhancing therapeutic specificity. **Type II RIPs:** Efficient entry and transport to ER can enhance the effectiveness of therapeutic applications.	**Type I RIPs:** Entry is less efficient; unclear exact mechanism of entry. **Type II RIPs:** Requires retrograde transport which may complicate cellular delivery and efficacy.	(35, 41)
Induction of Apoptosis	Apoptosis involves caspases (initiator, executioner, cytokine processor). Pathways include mitochondrial, death receptor, and endoplasmic reticulum stress. **Apoptosis-inducing factor** **(AIF):** Caspase-independent mechanism.	**Caspase Pathways:** Wellcharacterized pathways for inducing apoptosis in target cells. **AIF Pathway:** Provides an alternative apoptosis mechanism when caspase pathways are inhibited.	**Caspase Pathways:** Complex and involves multiple signaling pathways; might be influenced by cellular conditions. **AIF Pathway:** Less understood compared to caspase-dependent pathways.	(29, 42)
MitochondriaMediated Apoptosis	Induced by excessive reactive oxygen species, Ca2+ imbalance; leads to mitochondrial membrane potential changes, cytochrome c release, and activation of caspases. **Effects:** Changes in Bcl-2/Bax ratio, membrane permeability.	**Mitochondrial Pathway:** Effective in triggering apoptosis; well-documented in various studies. **Cytochrome c Release:** Key event in apoptosis and target for therapeutic intervention.	**Mitochondrial** **Pathway:** Variability in response among different cell types. **Complexity:** Involves multiple factors and steps that might lead to inconsistent results in different experimental conditions.	(42, 45, 46)
Death ReceptorMediated Apoptosis	Activated by receptors like Fas binding to FasL, leading to caspase-8 activation, which in turn activates caspase-3. **Example:** Marmorin triggers this pathway.	**Death Receptor Pathway:** Direct and well-defined mechanism for inducing apoptosis. **Selective Activation:** Can target specific apoptotic pathways in cancer cells.	**Variability:** Different RIPs may not affect all death receptor pathways equally. **Resistance:** Some cells or tumors may develop resistance to death receptor-mediated apoptosis.	(43)
Endoplasmic Reticulum StressMediated Apoptosis	Induced by misfolded protein aggregation, Ca^2+^ imbalance. Involves unfolded protein response (UPR) and proteins like Bip, CHOP, caspase4/12. **Examples:** Trichosanthin, marmorin.	**ER Stress Pathway:** Can target tumors by exploiting stress responses. **UPR:** Provides a dual approach by either prolonging stress to induce apoptosis or blocking UPR to enhance vulnerability.	**Complex Regulation:** UPR’s role in apoptosis can be complex and context-dependent. **Threshold Effects:** Some RIPs (e.g., α-MMC) require higher concentrations to be effective, which may limit therapeutic applicability.	(43, 47)

Bold values indicate key mechanistic pathways or molecular targets specifically associated with either Type I or Type II ribosome-inactivating proteins (RIPs) in their apoptotic activity. These distinctions highlight differences in pathway activation between RIP types (e.g., mitochondrial-mediated vs. ER stress-induced apoptosis) and emphasize critical regulatory nodes (e.g., caspase activation, NF-κB inhibition) central to RIP-induced cell death.

##### 3.2.3.1 Biological barriers in RIPs delivery

**Figure 2:**
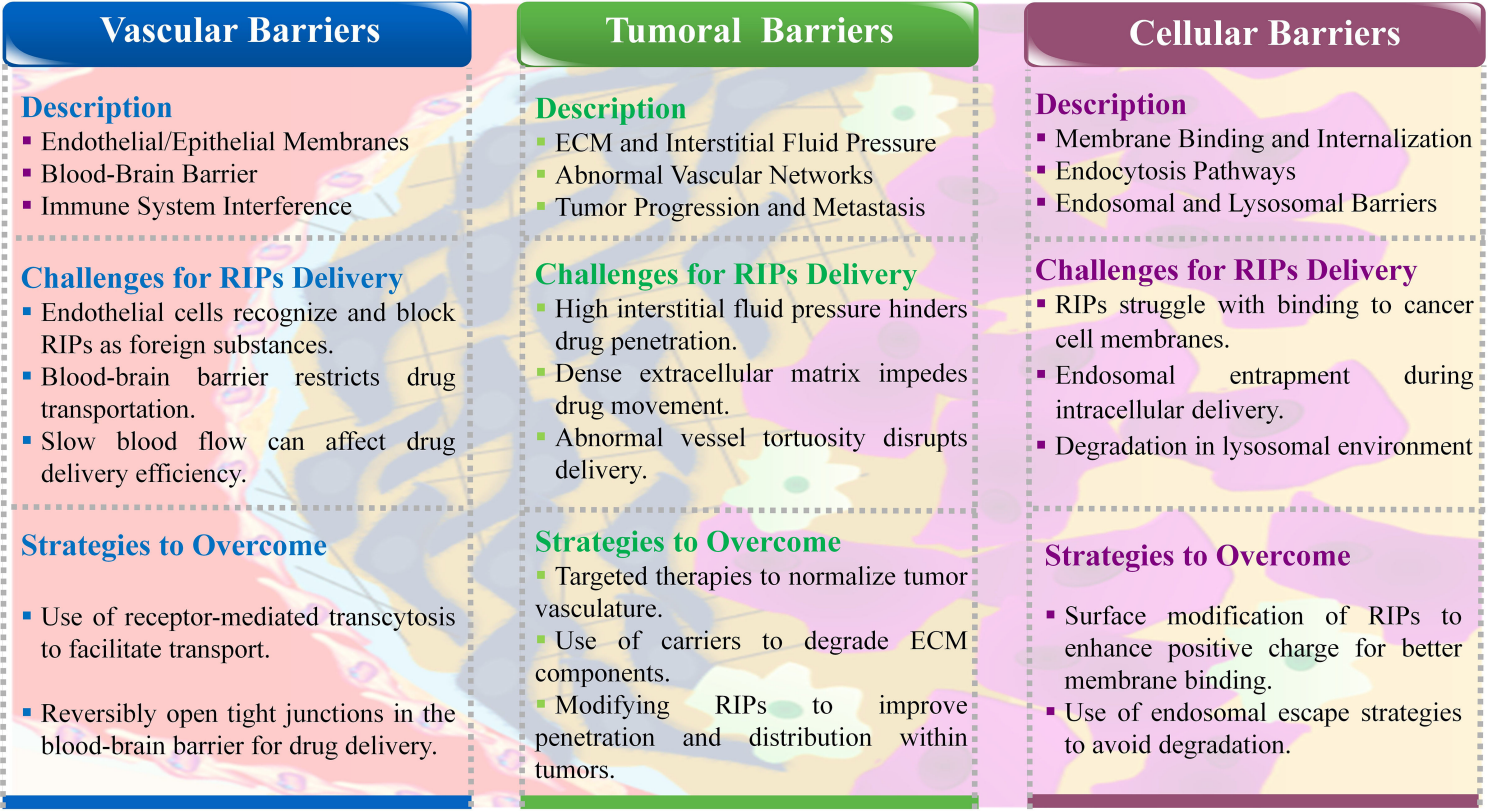
Schematic representation of biological barriers in RIPs delivery.

RIPs face significant challenges from the body’s bio-barriers when attempting to reach tumor sites. These problems include endothelial and epithelial cell membranes, the reticuloendothelial system, complex blood vessel networks, abnormal blood flow, and the high interstitial pressure within tumors. Additionally, the tumor cell membranes themselves pose a final barrier, hindering the transfection of RIPs into the cells. Overcoming these barriers is crucial for the effective delivery of RIPs and other anticancer therapies. Therefore, developing strategies to bypass these bio-barriers is essential for advancing protein-based cancer treatments (10). During the journey from the administration site to the target location, drugs face numerous biological barriers. Below, we provide a brief overview of these obstacles (**Figure 2**).

#### 3.2.4 Vascular bio-barriers

The body’s vascular system, including endothelial and epithelial cell membranes, acts as a barrier that prevents RIPs from reaching tumor sites. The selective permeability of these membranes recognizes RIPs as foreign agents, thus blocking their passage (48). The blood-brain barrier (BBB) is a particularly challenging obstacle, controlling the transport of essential nutrients and oxygen into the brain. For RIPs to cross the BBB, they must either exploit specific receptors or navigate through tight junctions between endothelial cells (49). Additionally, factors such as slow blood flow and the presence of immune cells can further impede the delivery of RIPs by deactivating the drug molecules, leading to reduced therapeutic efficacy (50).

#### 3.2.5 Intratumoral bio-barriers

Once RIPs reach the tumor vicinity, they encounter the dense extracellular matrix and high interstitial fluid pressure within the tumor, which further obstructs their penetration. Tumors often have disorganized and leaky vascular networks, leading to abnormal blood flow and pressure gradients that complicate drug delivery. These conditions create a physical barrier that prevents RIPs from effectively reaching and accumulating within the tumor (51). The extracellular matrix, which becomes denser and stiffer as the tumor grows, also plays a critical role in limiting drug penetration, thereby increasing the risk of metastasis and reducing the overall effectiveness of the therapy (52).

#### 3.2.6 Intracellular bio-barriers

Even after navigating through vascular and tumoral barriers, RIPs must overcome cellular barriers to exert their therapeutic effects. The cellular membrane, with its negative charge, can resist the entry of positively charged nanoparticles and large molecules like RIPs. Endocytosis, the primary pathway for cellular uptake, is size-dependent and may involve different mechanisms, such as clathrin-mediated or caveolae-mediated endocytosis. However, once inside the cell, RIPs may face degradation within the harsh lysosomal environment, which can prevent them from reaching their target in the cytosol and thereby reduce their ability to kill tumor cells (53).

In summary, RIPs face multiple bio-barriers at various stages of delivery, from vascular transport to cellular entry. Overcoming these barriers is critical for improving the delivery efficiency and therapeutic outcomes of RIP-based cancer treatments. Developing innovative strategies to bypass or mitigate these obstacles remains a key focus in advancing protein-based cancer therapies (10).

## 4 Delivery strategies of RIPs for improving anti-cancer activity

The delivery of drugs from their administration site to their target location is hindered by numerous biological barriers. Among these, intracellular barriers pose a significant challenge in the effective delivery of cytotoxic proteins. To address this, researchers have explored various approaches and proposed innovative systems to overcome this limitation. One such advancement is the use of the TAT peptide, a widely utilized cell-penetrating peptide (CPP) in drug delivery, which demonstrates the ability to transport a range of cargoes, including small molecules, proteins, genes, and nanoparticles, into virtually all cell types. Despite its versatility, the nonselective nature of TAT-mediated cell internalization presents a challenge in ensuring that the drug remains inactive and safe during circulation, while selectively activating it at the tumor site (10). To address these limitations, several advanced strategies have been developed, including: the Antibody Targeted Triggered Electrically Modified Prodrug Type Strategy (ATTEMPTS), enzymetriggered systems, pH-sensitive and charge-sensitive systems, nanotechnology-based approaches, light-activated internalization systems and Voltage-Gated Channel Targeting. **Table 2** summarizes the mechanism of each method, highlighting its advantages and disadvantages.

**Table 2:** Strategies for enhancing the efficacy of RIP in cancer treatment.

Strategy	Method/Mechanism	Advantages	Disadvantages	Key examples/Details	Ref.
Cell Penetrating Peptides (CPP)-Modified RIP Delivery	CPPs such as TAT peptide or low- molecular-weight protamine (LMWP) are conjugated to RIPs to facilitate membrane penetration and cellular uptake.	Enhanced intracellular delivery of RIPs, bypassing barriers like the plasma membrane. Increased cytotoxicity against cancer cells.	Non-selectivity can lead to off-target uptake, which may cause systemic toxicity.	TAT-gelonin and LMWP-modified gelonin showed 20–120-fold increased cytotoxicity in cancer cells compared to unmodified proteins. For instance, Gel-chlorotoxin fusion improved tumor targeting.	(10)
ATTEMPTS System	Combines tumortargeting ligands (e.g., T84.66 antibody) conjugated to heparin (negatively charged) with CPP-modified RIPs.	Controlled release of RIPs triggered by competitive interactions (e.g., protamine substituting CPPs). Reduced immune response due to heparin masking.	Requires complex design and optimization. Adding heparin can reduce cellular uptake efficiency of RIPs.	T84.66heparin/TAT-gelonin complex demonstrated selective cytotoxicity against carcinoembryonic antigen (CEA) positive colon cancer cells. Effective in both *in vitro* and *in vivo* colon cancer models.	(54)
EnzymeTriggered Delivery	Employs enzymesensitive linkers (e.g., matrix metalloproteinase-2responsive peptides) to selectively release RIPs in tumor sites.	Selective activation of RIPs at the tumor site minimizes systemic toxicity. Prodruglike stability in circulation.	Effectiveness depends on the specific enzyme expression in the tumor microenvironment. Linker degradation may vary between individuals.	PEGylated TCS linked with MMP-2-responsive peptides improved tumor-specific delivery and stability. Enhanced plasma circulation and reduced systemic toxicity in mouse models.	(55)
pH-Responsive Systems	Uses pH-sensitive polymers or proteins that activate or release RIPs in acidic tumor environments.	Effective targeting in tumors with acidic extracellular microenvironments. Reduces off-target effects in normal tissues.	May not function well in tumors with less acidic conditions. Sensitivity of the system to pH fluctuations requires optimization.	Stearoyl-PEG-poly SDM-modified liposomes demonstrated enhanced delivery in acidic environments.	(56)
Nanomaterial-Based Delivery	RIPs are encapsulated or conjugated with nanoparticles (e.g.,gold, liposomes) for improved delivery and protection.	Increased drug stability and circulation time. Enhanced tumor accumulation. Potential for codelivery of multiple drugs	Long-term toxicity of some nanoparticles remains a concern. Large-scale production and reproducibility can be challenging.	Silver nanoparticles conjugated with Gel showed enhanced delivery in melanoma models. Liposomal formulations of Gel with pHsensitive coatings improved stability and selective uptake.	(57, 58)
Light-Induced Systems	Uses photosensitizers that generate reactive oxygen species (ROS) upon light activation, disrupting endosomes and releasing RIPs.	Offers precise spatial and temporal control over drug release, minimizes off-target effects, and enhances cytosolic delivery.	Requires an external light source, limiting application to accessible tumors. Penetration depth of light is a limitation.	Gel/photosensitizer combinations demonstrated a 200-fold increase in cytotoxicity against cervical carcinoma cells. VEGF121/rGel combined with light-induced therapy eradicated melanoma in animal models.	(59)
Voltage-Gated Channel Targeting	Peptide toxins (e.g., scorpion toxins, bee venom). Targeting voltagegated sodium channels.	High selectivity for specific cell types. Potential for minimal off-target effects.	Limited understanding of the role of voltagegated channels in tumor biology. Potential for toxicity if channel function is disrupted in normal cells.	Requires further research to fully understand the therapeutic potential	(53, 60)

## 5 Role of RIPs as tumor suppressors

### 5.1 Effects of RIPs on breast cancer

Several RIPs have been reported for their anti-cancer and anti-tumor activities. By activating caspase-8 and caspase in breast tumor cells, RIPs induce cell cycle arrest, decrease tumor growth and volume, and restrict cell survival. An immunotoxin is designed to kill various cells and solid tumors based on the RIP model. Superior structural and functional characteristics are found in Saporin-S6 immunoconjugates. Saporin-S6 is immune to proteolysis and denaturation, is highly catalytic, and causes less damage to healthy cells. Type I RIPs have been found to inhibit breast tumor cell proliferation *in vitro* and *in vivo* (61–63). Human epidermal growth factor receptor 2 (HER2) and fibroblast growth factor-inducible 14-kDa protein (Fn14) are frequently co-expressed in human breast tumors, and HER2 directly induces an increase in Fn14 expression, making tumor cells sensitive to an immunotoxin made by fusing Fn14 antibodies to recombinant gelonin (designated hSGZ) (64). Recombinant gelonin (rGel) can be enzymatically blocked from producing proteins in tumor cells thanks to hSGZ’s ability to rapidly internalize and distribute it to the cytoplasm. Since Fn14 promotes the migration and invasion of breast cancer cells, one may wonder if there is a way to harm tumor cells while simultaneously decreasing Fn14 expression. Since MAP30 can reduce HER2 expression, leading to decreased Fn14 expression, and hSGZ can target Fn14-positive cells and exert its function without increasing the invasive capacity of tumor cells, we hypothesize that using them together might achieve a better outcome, and breast tumor cells can be sequentially treated with MAP30 and hSGZ. However, further testing is needed to confirm this hypothesis. Many receptors on cell membranes are expressed at low levels by healthy cells but at high levels by cancerous ones. In contrast to normal breast tissues, tumor tissues express estrogen receptors (ER) at significantly greater levels in around 75% of breast cancer cases (P = 0.001) (29). The proliferation-inhibiting effects of marmorin in ER-positive breast cancer cells include the ER-mediated signaling system, which is the target of numerous medicines. It has been hypothesized that marmorin might starve tumors to death by reducing the number of blood vessels *in vivo* (65), due to its capacity to inhibit angiogenesis by reducing the survival of human umbilical vein endothelial cells *in vitro*. In mice with a xenograft of the MDA-MB-231 tumor, marmorin causes DNA damage and endoplasmic reticulum stress, leading to the activation of apoptosis (65). Although RIPs may one day be used as novel anti-tumor agents, they are not without serious side effects, such as anaphylaxis, immunogenicity, and toxicity that can affect the entire body. Deng et al. (66) investigated the anti-tumor efficacy of polyethylene glycol (PEG)-modified -MMC in breast carcinoma and found that PEGylation of -MMC increases the half-life of -MMC and reduces non-specific toxicity. There was a marked improvement in anti-tumor activity with a manageable level of adverse responses with -MMC-PEG.

A novel functional gelonin fusion protein, denoted as Trx-PVGLIG-pHLIP-gelonin (TPpG), was genetically engineered and characterized for the first time. TPpG is a multi-functional construct composed of four distinct components, each designed to fulfill a specific role in tumor targeting and therapy. The thioredoxin (Trx) tag enhances the solubility and stability of the fusion protein, while the MMP-2/9-cleavable peptide (PVGLIG) ensures responsiveness to the tumor microenvironment by being cleaved in the presence of overexpressed MMP-2/9 enzymes. The pHLIP (pH Low Insertion Peptide), with a specific sequence (AAEQNPIYWARYADWLFTTPLLLLDLALLVDADEGT), confers tumor acidity-targeting capability, and gelonin, a plant-derived ribosome-inactivating protein, serves as the cytotoxic agent (67).

Asim Pervaiz et al. (2023) investigated the deregulation of anticancer genes (NOXA, PAR-4, TRAIL) in breast cancer and their induction by riproximin, a ribosome-inactivating plant protein. Expression analysis in 45 clinical samples and two breast cancer cell lines showed NOXA deregulation, PAR-4 downregulation, and TRAIL upregulation, correlating with disease severity. Riproximin significantly induced these genes at transcriptomic and protein levels, highlighting its potential therapeutic role. Bioinformatics analysis confirmed their involvement in key cellular pathways. This study suggests riproximin as a promising agent for restoring anticancer gene expression in breast cancer (68).

Shen et al. developed a TME-responsive nanoplatform using a pH-sensitive polymer and a cationic lipid-like compound for cytotoxic saporin delivery in breast cancer therapy. This approach enhances intracellular uptake, promotes endosomal escape, and effectively inhibits tumor growth, highlighting its potential for targeted protein-based treatments (69).

### 5.2 Effects of RIPs on leukemia and lymphoma

TCS inhibits the proliferation of B-lymphocyte cell lines by arresting them in the S-phase of the cell cycle, and it causes death in T-lymphocyte cell lines (70). Cucurmosin (type I RIP) is said to be more effective than TCS at killing chronic myelogenous leukaemia K562 cells, despite the fact that both drugs suppress cell growth by downregulating P210Bcr-Ab1 and tyrosine kinase (71). Lymphoma and leukaemia may be inhibited by TCS’s anti-proliferative properties. Tlymphocyte cell death is induced by blocking the cell cycle in the S phase, while B-lymphocyte growth is stifled (72). Type 2 RIP ripoximin inhibited cell proliferation, apoptosis resistance, and motility by downregulating Rho GTPases in MDA-MB-231 and MCF-7 human cells. IL24/MDA7, an anti-cancer cytokine, and the ER stress-related GADD genes were both elevated by ripoximin (73). Curcin 1 of the ribosomal inhibitory protein family reduces tumor cell growth. T-cell leukaemia is one type of cancer that may be inhibited by viscumarticulatum RIP. Apoptosis is triggered alongside the elevation of mitochondrial potential via early articulating D signaling. Compared to RTA alone, RTA combined with anti-HER2 scFv 4D5 and the endoplasmic reticulum-targeting peptide KDEL was 440 times more effective against ovarian cancer cells (74). RIPs ITs have potent anti-cancer effects, especially against haematological diseases, which are more easily treatable than solid tumors. Improved ITs should be directed at the differentiation clusters that can be identified on the surface of haematological cells. Human lymphoma and leukaemia surface proteins CD7, CD2, CD19, and CD22 were used to create Sap-So6 (75–78).

The growth of many different types of leukemia and lymphoma cell lines is considerably slowed by trichosanthin. Cucurmosin and TCS inhibit tyrosine kinase, which in turn suppresses cell proliferation by downregulating P210Bcr-Ab1 (79).

Several lines of leukemia and lymphoma cells are sensitive to TCS’s anti-proliferative effects (80). Importantly, TCS can cause damage to leukemia and lymphoma cells in distinct ways. TCS suppresses the proliferation of B-lymphocyte cell lines by causing them to enter an S-phase cell cycle arrest, while it promotes apoptosis in T-lymphocyte cell lines (80). It has been hypothesized that cucurmosin is more effective than TCS at killing chronic myelogenous leukemia K562 cells, although both compounds decrease cell proliferation by downregulating P210Bcr-Ab1 and tyrosine kinase (71). It has been found that the combination of cucurmosin plus trans-retinoic acid or arsenic trioxide synergistically increases these effects on the human acute promyelocytic leukemia NB4 cell line (81).

The cytotoxicity of Articulatin-D, the first cytotoxic RIP with a B-chain missing sugar-binding function, was demonstrated *in vitro* against a variety of leukemia and lymphoma cell lines, with Jurkat cells showing the highest sensitivity, followed by Molt-4, U-937, HL-60, and Raji cells (82). Articulatin-D’s unique physical features make it a promising starting point for the development of immunotoxins that can target and kill tumor cells with pinpoint accuracy. Emerging immunotoxins combine a toxin fragment with an antibody or cytokine to create a specific therapeutic agent. Several studies (83–85) and clinical evaluations (86–88) have reported the efficacy of immunotoxins made from saporin and rGel in the treatment of cancer. Cancer cells may be located by their membrane proteins CD22, CD7, CD19, and CD38, and the immunotoxins built against them use the corresponding antibodies HB22.7, HB2, BU12, and OKT10. Cytotoxic activity of HB22.7-saporin against a panel of non-Hodgkin’s lymphoma (NHL) cell lines and considerable inhibition of tumor growth in a xenograft model of NHL was demonstrated (88). Selective cytotoxicity against human acute lymphoblastic leukemia has been demonstrated *in vitro* and *in vivo* (89) using HB2-saporin, BU12-saporin, and OKT10-saporin. Treatment with rGelBLyS, rGel fused to a B-lymphocyte stimulator, significantly increased survival in a mouse model of disseminated lymphoma or leukemia (xenograft model) and significantly decreased tumor burden; in this context, cell death was not induced by caspase activation but was partially mediated by the ribotoxic stress response, as reported by Luster et al. (90) Nuclear factor kappa B (NF-B) activity, essential for cellular proliferation and survival, was inhibited by the combination of the rGel-BLyS fusion toxin and the proteasome inhibitor bortezomib (91).

### 5.3 Effects of RIPs on other cancers

Immunotoxins, made from substances like saporin and rGel, are effective against cancer. In a xenograft model of NHL, HB22.7-saporin was demonstrated to dramatically inhibit tumor growth. It was also cytotoxic against a panel of non-Hodgkin’s lymphoma (NHL) cell lines (92). RIP’s Impact on Other Forms of Cancer Recent studies in cell culture and mice have shown that MAP30 has anticancer action. For instance, MAP30 decreased cell viability and S Phase arrest in a dose- and time-dependent manner in HepG2 cells. Tumor volume was also reduced in HepG2-bearing mice as a result of MAP30-induced apoptosis and necrosis. In HepG2 cells, cucurmosin triggered G0/G1 cell cycle arrest and death, which translated to potent anti-tumor action *in vivo*. Abrus agglutinin induces cascade activation and inhibits act phosphorylation and NF-B expression in HepG2 cells (93). Furthermore, Luffa cylindrica recombinant luffin exhibits cytotoxicity *in vitro* against several tumor cell lines. In a dose- and time-dependent way, recombinant luffin suppressed the growth of JEG-3 (human placental choriocarcinoma), HepG2 (human hepatoma), and MCF-7 (human breast cancer) cell lines (94). Trichosanthes kirilowii’s tianhua (TH-R) has been shown to suppress the growth of the human lung cancer A549 cell line by dose- and time-dependently interrupting the G0/G1 phase of the cell cycle and inducing apoptosis (95). Cell survival, cell proliferation, angiogenesis, and inflammation are just some of the cellular processes that have been demonstrated to be regulated by fibroblast growth factor-inducible 14 (Fn14), a member of the tumor necrosis factor (TNF) receptor superfamily. Fn14 is expressed locally at significantly higher levels in wounded and diseased tissue (96), despite being expressed at relatively low levels in normal tissues. Highly cytotoxic to an FN14-expressing tumor cell line was recombinant gelonin (rGel), a type I RIP conjugated to an anti-Fn14 monoclonal antibody (ITEM-4). Nude mice carrying a xenograft of T-24 human bladder cancer cells showed improved long-term tumor growth inhibition after receiving an immunoconjugate with ITEM-4-rGel (97). Hepatoma and other malignancies in relation to RIPs. Anti-tumor effect of MAP30 was demonstrated in cell culture and mice. Time- and dose-dependent MAP30 inhibition of cell viability and S-phase arrest were observed in HepG2 cells; furthermore, MAP30-induced apoptosis and necrosis resulted in tumor volume decrease in HepG2-bearing mice (98). These effects of cucurmosin translated into strong anti-tumor properties *in vivo* (99), and G0/G1 arrest and death were triggered in HepG2 cells. Abrus agglutinin inhibits Akt phosphorylation and NF-B expression in HepG2 cells (100), and it also triggers the caspase cascade.

Morrison and colleagues developed a bioinspired delivery platform to enhance the therapeutic potential of saporin, a ribosome-inactivating plant toxin with poor membrane permeability. Using a pH-responsive pseudopeptide, poly (L-lysine isophthalamide) grafted with 50% L-phenylalanine (PP50), they significantly improved saporin’s cytotoxicity in A549 lung cancer cells and 3D spheroids under mildly acidic conditions. PP50/saporin inhibited protein synthesis, activated caspases 3/7, 8, and 9, and induced apoptosis via micropinocytosis and caveolae-mediated endocytosis. Fluorescent labeling confirmed saporin delivery to the cytoplasm and nucleus. These findings establish PP50 as a promising platform for intracellular delivery of protein therapeutics, enhancing cancer treatment efficacy (101).

In the study in 2023 analyzed the expression of anticancer genes (NOXA, PAR-4, TRAIL) in colorectal cancer (CRC) and their induction by riproximin, a ribosomal-inactivating plant protein. Their study revealed a consistent downregulation of anticancer genes in CRC, with NOXA and PAR-4 significantly suppressed during liver metastasis, while TRAIL showed early induction. Riproximin effectively upregulated these genes in CRC cell lines, suggesting its potential as a therapeutic agent (102).

In 2024, the research team investigated a novel antitumor strategy by exploring the synergistic effects of QS-21, a cationic amphiphilic saponin, and MAP30, a RIP, in inducing lysosomedependent cell death (LDCD). Their study highlights that while QS-21 alone induces lysosomal membrane permeabilization (LMP) without cytotoxicity, its combination with MAP30 significantly enhances apoptosis in tumor cells at low concentrations. Mechanistically, QS-21 facilitates the escape of MAP30 from lysosomes into the endoplasmic reticulum, where MAP30 suppresses lysophagy by downregulating LC3 proteins. This inhibition prevents the clearance of damaged lysosomes, leading to the accumulation of lysosomal contents such as cathepsins in the cytoplasm, ultimately triggering LDCD. The findings suggest that the coadministration of QS-21 and MAP30 could serve as a promising synergistic antitumor approach by amplifying lysosomal disruption (103).

Ren et al. (2025) developed optimized lipid nanoparticles (LNPs) to enhance protein delivery for cancer immunotherapy.These LNPs efficiently delivered various therapeutic proteins, including saporin and IL-10, which inhibited tumor growth and enhanced T cell responses in melanoma models. This study highlights the potential of LNPs for advancing protein-based therapies in cancer treatment (104).

### 5.4 Challenges and future directions

The therapeutic potential of RIPs, particularly in anti-HIV and anti-cancer applications, has gained attention over the past decades (105). Despite this, RIPs have been used in clinical trials as part of immunotoxins to treat malignant tumors (106). RIPs face several challenges in therapeutic applications. Despite their immunosuppressive activity, plant-derived RIPs often trigger immune responses, potentially leading to allergic reactions (35). Additionally, their short plasma half-life limits exposure to target cells, necessitating repeated administration, which in turn increases immune reactions. Furthermore, neurotoxicity has been observed in HIV-infected patients treated with RIPs like TCS, and accessing solid tumor masses presents a significant obstacle to effectiveness (107). Advances such as PEGylation have been explored to improve RIPs’ pharmacological properties by increasing their half-life and reducing antigenicity, with the potential to enhance tumor targeting (69). Although PEGylation has extended plasma half-life and reduced antigenicity, challenges remain, such as non-selective cytotoxicity. Immunotoxins combined with chemotherapy or other ITs offer promising strategies to enhance efficacy while minimizing side effects. Studies have shown that combining RIP-based ITs can improve outcomes, as seen in clinical trials involving treatments for acute graft-versus-host disease and lung cancer (105). However, severe side effects have led to the failure of some RIP-based drugs in clinical trials. New techniques like photochemical internalization and conjugation with albumin-binding peptides provide promising avenues for future research and development (105).

Current cancer therapeutics often lack precise tumor specificity, resulting in limited efficacy and off-target effects that can compromise treatment outcomes. Targeted therapies, such as immunotoxins with bispecific antibodies or tumor-specific expression of RIPs, offer potential solutions. These strategies must undergo extensive preclinical studies to evaluate their safety and effectiveness. Factors like immunogenicity, toxicity, and dosage must be considered when translating preclinical findings to clinical applications. While saporin-containing immunotoxins have shown promise in clinical trials, more research is needed to refine tumor-specific expression strategies and enhance RIP-based therapies (106).

As mentioned in the first section, cancer presents formidable challenges to treatment due to its diverse nature, rapid development of therapeutic resistance, and the complexity of its cellular pathways. In the treatment of cancer, the diverse nature of the disease and its rapid resistance to therapies present significant obstacles for single-agent approaches like RIPs. While RIPs exhibit potent anti-cancer effects, their toxicity and lack of specificity pose challenges to their clinical application. To overcome these limitations, combining RIPs with MSCs offers a promising therapeutic strategy. MSCs possess unique tumor-targeting properties and can serve as carriers for RIPs, allowing for more precise delivery of the cytotoxic proteins directly to the tumor site, minimizing off-target effects. Furthermore, MSCs have immunomodulatory capabilities, which can help reduce the immune responses triggered by RIPs, improving the safety profile of this combination therapy. Unlike MSCs, which have the potential to differentiate into undesired cell types or contribute to tumor progression under certain conditions, EVs lack the ability to proliferate or transform, reducing safety concerns. MSCs and their EVs offer a complementary mechanism by leveraging their intrinsic tumor-tropism. MSCs can migrate to tumor sites, delivering therapeutic agents directly to the microenvironment. However, their use raises concerns about their potential to promote tumor growth or metastasis under certain conditions. In contrast, MSCderived EVs circumvent many of these risks while retaining the therapeutic benefits (108).

By leveraging the tumor-homing ability of MSCs and the potent anti-cancer activity of RIPs, this combination could significantly enhance therapeutic efficacy while reducing systemic toxicity. As a potential hypothesis, this synergistic approach could be tested to evaluate how well it addresses the challenges of cancer treatment, including drug resistance and therapeutic targeting.

In the next section, we will explore the advantages, disadvantages, and challenges of MSCs to develop strategy for cancer therapy and advantages of MSC-derived EVs over MCSs. Next, we explore the possibility of combining these two treatment strategies.

## 6 MSCs and their dual role in cancer therapy

MSCs are multipotent stem cells known for their high differentiation potential and self-renewal capabilities. Initially identified in adult bone marrow in the late 1960s, MSCs were later named for their ability to differentiate into various cell types (109–112). So far, MSCs have been isolated from various tissues including bone marrow and adipose tissue, umbilical cord, peripheral blood, dermis, liver, epidermis, and skeletal muscle, etc. (14, 18, 109, 113).

The reciprocal interactions between cancer cells and the non-cancerous stromal and immune cells within the TME are essential in the pathophysiology of cancer. Factors secreted by tumor cells influence the tumor-tropism, reprogramming, and fate of MSCs, while MSCs, in turn, can impact the fate and progression of cancer cells through the secretion of chemokines and cytokines. MSCs secrete various effector molecules that mediate their dual roles in either supporting or suppressing tumor growth. Regardless of their origin, MSCs play a critical role in the TME, contributing to either tumor-suppressive or tumor-supportive conditions (114).

Over the past two decades, MSCs have been widely used in regenerative medicine and cancer treatment due to their ability to influence tumor behavior and their tumor-targeting properties. However, despite these promising traits, MSCs have also been found to support tumor growth in some cases. These conflicting findings highlight the need for careful consideration of MSCs’ complex biological characteristics in therapeutic applications to prevent unintended consequences (13).

**Figure 3:**
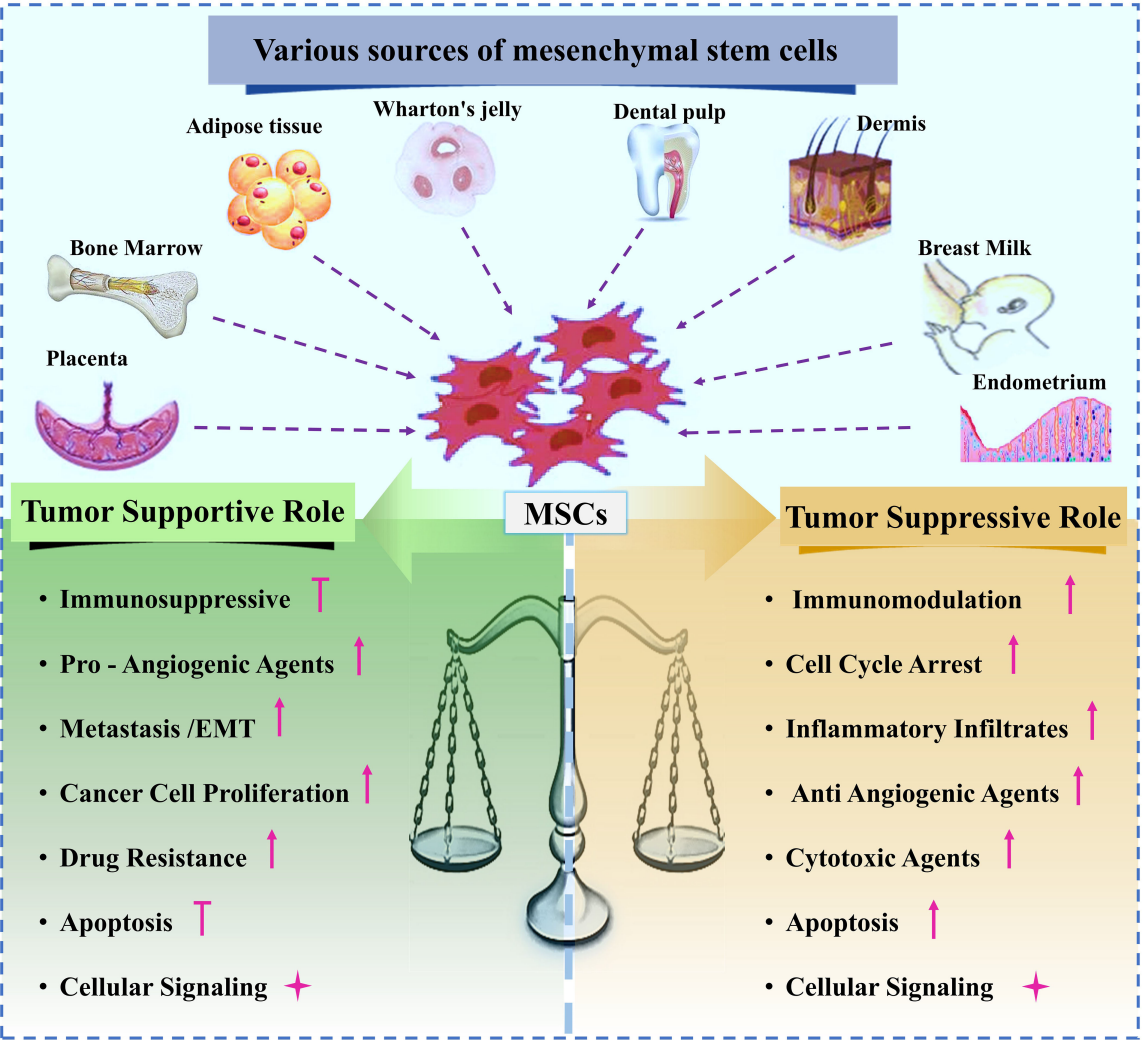
Various sources of MSCs, and dual effect of MSC on tumor progression.

These dual and often contradictory functions must be carefully considered when therapeutic MSCs are used, as they may adopt tumor-supportive roles within the TME, similar to tumorassociated MSCs (TA-MSCs). The subsequent sections will explore the different pathways through which MSCs can exert either tumor-suppressive or tumor-supportive effects (13) Various sources of MSCs and their dual roles in either supporting or suppressing tumor growth are schematically shown in **Figure 3**.

### 6.1 Tumor-supportive properties of MSCs

Cancer progression is affected by the TME, which consists of stroma, non-cancerous cells, and malignant cells. Multiple cell groups might be present in the extracellular matrix and tumor stroma. Fibroblasts, endothelial cells, and myofibroblasts are some of the many kinds of immune cells. After reaching tumor sites, MSCs join the stroma (115). Tumor growth is stimulated when these cells engage with cancer cells. Evidence from studies using co-implanted cancer cells (116) and mouse models of breast cancer (117) showed that MSCs may increase tumor growth and metastasis. MSCs are drawn to tumor where they can aid in the growth and spread of cancer (118, 119). Receptors and chemokines that encourage the migration of other support cells to tumors are likely mediators of the processes that allow MSCs to home (120). These include angiogenic factors (FGF, HIF1, and VEGF), chemokines (CCL5, CCL2, CXCL12, and CCL22), and inflammation factors (TGF, TNF, IL-8, and IL-1) (121, 122). Recruiting MSCs to different malignancies may have a significant effect on the course of the tumor -Metastasis and tumor -promoting MSCs were demonstrated in a rodent model of breast cancer (123). Here, we investigate the impact of MSCs on the growth of tumor. The supportive mechanisms of MSCs on tumor growth are presented in **Table 3**.

**Table 3:** The Role of MSCs in supporting and suppressing tumor growth.

Function	Effect	MSC-derived molecule	Impact on tumor growth	References
Tumor supportive	Immunosuppression	TGFβ, IFNγ, TNFα, PGE2, CCL2, galectin-9, HGF, CTLA-4, soluble PD-L1 and PD-L2, NO, HLA-G, IDO, IL-1α, IL-1β, IL-4 and IL-6	Suppresses T cell and B cell proliferation, NK cell activity, DC maturation	(109, 124, 125)
	Stimulation of Angiogenesis	IL-1α, IL-6, TGF-β, VEGF, IGF, EGF, FGF, PDGF, MFG-E8, Artemin, Axl, Osteoprotegerin, Angiopoietin-like Factor, MIP 2, Ang1, SDF-1/CXCL12	Promotes tumor blood vessel formation, recruits MSCs to neovascularization sites	(126–128)
	Epithelialmesenchymal transition	HGF, EGF, PDGF, leptin and TGFβ	Induces EMT, increases metastatic capacity	(129, 130)
	Correlation with cancer stem cells	BMP, IL-6, IL-8, CXCL6, and CXCL5	Increases proliferation and invasive properties of CSCs	(131, 132)
	Tumor metastasis promotion	Lysyl oxidase (LOX), TGFβ, FGF, HGF, EGF, CCL5, CXCL5, CXCL1, CXCL7 and CXCL8	Promotes tumor cell migration, enhances tumor cell invasiveness	(133, 134)
	Inhibits apoptosis	L-8, Prosaposin (PSAP), Thymosin beta 4 X-linked (TMSB4X), CCL- 5, CXCL-1, IP-10, MSCP-1, Prostaglandin E2 (PGE2), miR- 30a, miR-21-5p, miR-222, Long non-coding RNA, PVT1.	Promotes tumor proliferation	(13)
	Promotes drug resistance	CXCL12, EGF, IGF, IL-6, IL-7, IL-8 and PGE-2	Reduces caspase 3 activity, inhibits apoptosis	(135, 136)
Tumor suppressive	Inhibition of angiogenesis	Extracellular vesicles (EVs), cytokines (e.g., IL-10, TGF-β), anti-angiogenic factors (e.g., endostatin, thrombospondin)	Regulating key pathways such as Wnt/β-catenin and PDGF/PDGFR	(126, 137)
	Inhibition of metastasis	EVs, cytokines (e.g., IL-10, TGF- β), TIMP-1, TIMP-2 and TIMP-3, miR-3940-5p, miR-21-5p	Modulate TME to suppress metastatic behavior	(138, 139)
	Inhibition of tumor progression and Induction of apoptosis	Dickkopf-1 (DKK-1), IL-28, TIMP-1, CINC-1, miR-15a-5p	Regulating cell cycle genes and promoting cellular senescence through caspase-3 and BAX signaling pathways	(13, 140)
	Regulation of Immunologic Balance	Cytokines (e.g., IL-10, TGF-β), EVs	Expression level of FoxP3 in naive T CD4 + cells, and promote the TGF-β-Smad2 signaling pathway	(141)

### 6.2 Tumor-suppressive properties of MSCs

While many studies have shown that MSCs promote tumor growth, others have shown that they inhibit tumor growth (**Figure 3**). As a result, mesenchymal stem cells (MSCs) are thought to inhibit tumor growth by increasing inflammatory cell infiltration (142), stopping angiogenesis (143), decreasing Wnt and AKT signaling (144, 145), causing cell cycle arrest and death, and preventing angiogenesis (146, 147). MSCs isolated from adipose tissue secreted interferon-, which inhibited the growth of MCF-7 cells, according to recent studies by Ryu et al. (148). In addition, MSCs primed with IFN- or cultured in 3D systems can secrete TRAIL, causing tumor-cell-specific apoptosis (149). The primary mechanisms by which MSCs exert their tumor-suppressive effects are summarized in this section (**Table 3**).

### 6.3 Signaling pathways regulated by MSCs

**Figure 4:**
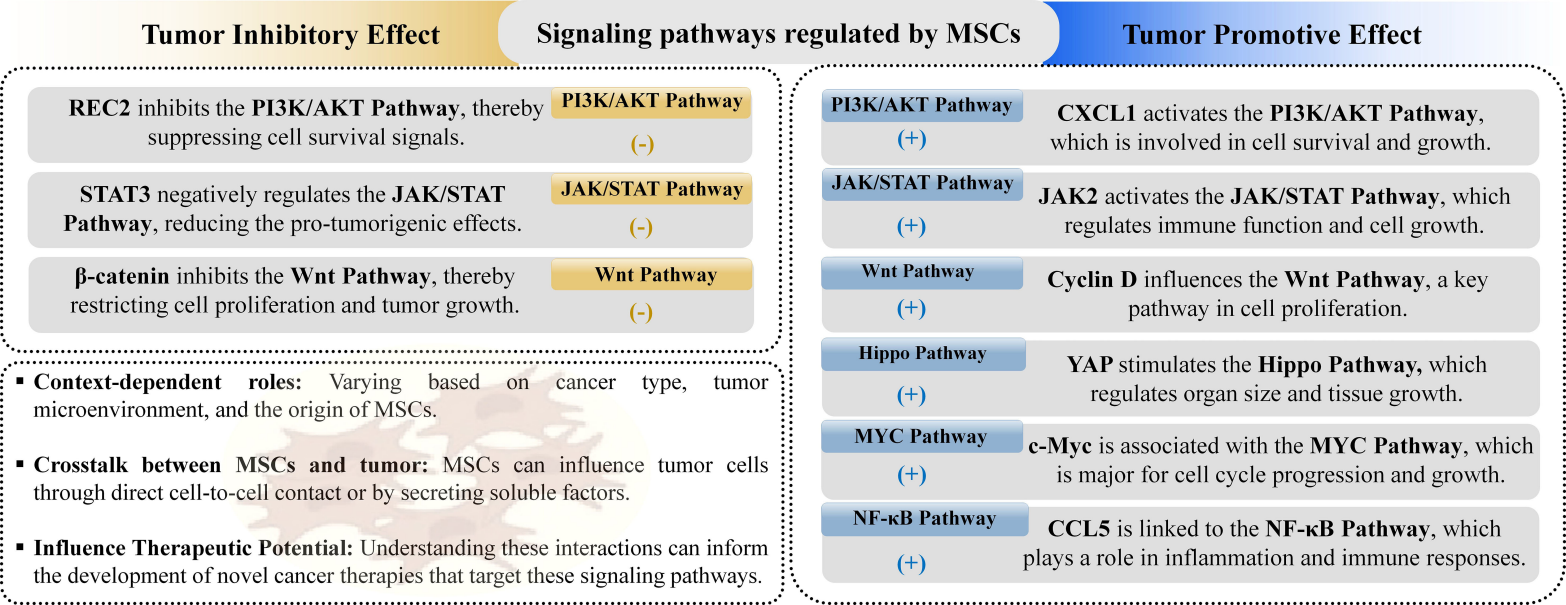
MSCs influence cancer-related signaling pathways, playing both tumor-promoting and tumorsuppressing roles. They achieve this by either upregulating or downregulating specific pathways involved in tumor growth and inhibition.

Cancer progression is intricately linked to various signaling pathways, which are modulated by MSCs in ways that can influence tumor growth. Key pathways affected by MSCs include PI3K/AKT, JAK/STAT, Wnt, Hippo, MYC, and NF-κB (**Figure 4**).

Among these pathways, the PI3K/AKT pathway is notably significant, as its aberrant activation or inhibition is frequently observed in various cancers, contributing to increased cell proliferation, drug resistance, and stem-cell-like phenotypes (150). PI3K, activated by numerous upstream factors, and AKT, a key downstream effector, are involved in multiple signaling cascades that influence cancer development (151). Evidence suggests that MSCs can affect tumor growth and metastasis through the PI3K/AKT pathway by promoting or inhibiting various cancer processes. For instance, MSCs associated with breast cancer can enhance mammosphere formation and create a tumor-friendly environment via this pathway, while MSC-conditioned media has been shown to boost the progression of head and neck cancer (152, 153). The JAK/STAT signaling pathway, another evolutionarily conserved cascade, plays a important role in regulating growth factors and cytokines, impacting various tissue development trajectories and disease progression, including cancer. Dysregulation of this pathway has been linked to tumorigenesis, with specific STAT proteins contributing to cancer cell malignancy (154). Studies on MSC interactions with tumors via JAK/STAT pathways reveal mixed roles. For example, IL-6 secreted by MSCs in colorectal cancer can activate JAK2/STAT3 signaling (155), promoting cancer progression (156), whereas MSC-conditioned media has been reported to inhibit STAT3 levels in breast cancer, suggesting potential anti-tumor effects (157). The Wnt signaling pathway, crucial for tissue development and cancer regulation, exhibits complex interactions with MSCs, with some studies showing MSCs modulating Wnt signaling to enhance cancer cell proliferation and metastasis, while others indicate that MSCs secrete Wnt antagonists to inhibit cancer growth (158). Similarly, the Hippo signaling pathway, involved in regulating cell growth and organ development, has been shown to influence cancer progression when modulated by MSCs, although evidence is limited and further investigation is needed (159). The MYC signaling pathway, associated with oncogenesis and poor prognosis in various cancers, is influenced by MSCs that can either promote tumor growth or enhance drug resistance (158). Lastly, the NF-κB pathway, a critical regulator in cancer progression, is implicated in MSC-mediated pro-tumor effects through its involvement in stemness and chemoresistance. Overall, these findings underscore the intricate role of MSCs in cancer signaling pathways, highlighting the need for targeted research to elucidate their therapeutic potential and impact on tumor biology (160).

### 6.4 MSC-derived EVs and their role in cancer therapy

A major challenge facing potential therapeutic applications of MSCs lies in their safety profile. MSCs are known to play a dual role in immune regulation; while they exhibit immunosuppressive properties in certain contexts, they can also act as antigen-presenting cells, thereby triggering immune responses. Additionally, MSCs carry a risk of tumorigenicity, either through their direct malignant transformation or by indirectly promoting the growth of existing tumor cells. Evidence indicates that MSCs are present in several tumor types, such as gastric adenocarcinoma, lipoma, and osteosarcoma, underscoring their potential role in tumor development. Consequently, it is crucial to carefully evaluate the risks and benefits of MSC-based therapies for each patient (161).

In recent years, there has been growing interest in the use of EVs derived from MSCs (MSCEVs). These EVs act as carriers of bioactive molecules, such as RNAs and proteins, transferring their cargo from parent cells to target cells and mimicking the biological functions of their origin cells. Emerging evidence suggests that MSC-EVs retain the therapeutic properties of MSCs while mitigating the safety concerns associated with live cell therapies. As a result, MSC-EVs are increasingly being considered as a promising alternative to MSCs, paving the way for cell-free therapies in future clinical applications (162).

Efficient and scalable isolation of pure EVs is critical for advancing biological research and clinical applications. Various EVs isolation methods have been developed, including centrifugation, polymer precipitation, size-dependent separation, immunoaffinity capture, and microfluidic technologies. Centrifugation, the gold standard, is effective but time-intensive and yields low purity. Polymer precipitation, often using PEG, enhances EVs recovery but introduces polymer impurities. Size-dependent methods, such as ultrafiltration and size exclusion chromatography (SEC), offer better purity but lack specificity for EVs subtypes. Immunocapture selectively isolates EVs based on surface markers (e.g., CD9, CD63, CD81) but is limited by sample size and tumor heterogeneity. Emerging microfluidic technologies leverage EVs properties such as size, density, and immunoreactivity, offering advantages like high sensitivity, rapid processing, and cost-effectiveness while being compatible with conventional methods (163).

Accurate identification and quality control of EVs are essential for their use in biological research and clinical applications. Established methods such as electron microscopy, capillary electrophoresis, nano-plasmonic sensing dynamic light scattering, and nanoparticle tracking analysis enable characterization of EVs physical properties like size and morphology, although each has limitations related to sample preparation or measurement consistency (164). Omics analyses further reveal the molecular composition of EVs, highlighting their enrichment in noncoding RNAs (e.g., miRNAs, circRNAs, lncRNAs) and functional proteins. EVs proteins are categorized into membrane-associated proteins, cytoplasmic proteins, and contaminants from the purification process, with markers like CD90 and CD29 aiding MSC-EVs identification. Techniques like western blotting, ELISA, and flow cytometry further support EVs characterization. Despite advances, improved methodologies for EVs isolation and identification are critical to provide their therapeutic potential (165).

### 6.5 MSC-EVs and cancer therapy resistance

Therapy resistance represents one of the most significant challenges in clinical cancer treatment, contributing to higher recurrence rates and increased mortality. Understanding the underlying mechanisms of therapeutic resistance is essential for devising effective strategies to eradicate cancer and prevent relapse. Cancer therapy resistance is often attributed to several mechanisms, including rare pre-existing drug-resistant clones that drive recurrence and treatment failure; the adaptive responses of cancer cells, which involve phenotypic plasticity (e.g., transdifferentiation, dedifferentiation and metabolic alterations); and changes within the TME, such as the activation of cancer stem cells (166). Additionally, drug-specific mechanisms, such as enhanced drug efflux, inactivation, or modifications to drug targets, also play a role. To address these challenges, new approaches are needed to uncover the fundamental drivers of resistance and implement strategies to counteract them, thereby improving treatment efficacy. MSC-EVs have emerged as key players in cancer development, progression, and therapy resistance. Their potential as an anticancer modality for clinical use has been widely recognized. **Table 4** summarizes recent progress in understanding how MSC-EVs contribute to therapy resistance, with a particular focus on their roles in radiotherapy, chemotherapy, targeted therapy, immunotherapy, and endocrine therapy (165).

**Table 4:** Role of MSC-EVs in regulating cancer therapy resistance.

Mechanism of Resistance	Cancer Type	MSC Source	Cargo/Con tent in EVs	Target Pathway	Effect on Resistance	Ref.
Radiotherapy Resistance	Nasopharyngeal Carcinoma (NPC)	Bone Marrow MSCs	miR-34c	β-Catenin signaling	Inhibited proliferation, migration, and radiotherapy resistance. Increased radiationinduced apoptosis in NPC cells.	(167)
	Melanoma	Umbilic al Cord MSCs	EVs cargo (unspecified)	Radiationinduced apoptosis pathway	Enhanced radiotherapy response by reducing tumor growth and systemic spread.	(168)
Chemotherapy Resistance	Gastric Cancer	Umbilic al Cord MSCs	miR-301b3p	CaM- Ks/Raf/MEK/ ERK pathway	Promoted drug resistance to 5-FU and cisplatin by inhibiting apoptosis.	(169)
	Breast Cancer	Adipose - Derived MSCs	miR-1236	Wnt/β-Catenin signaling pathway	Inhibited drug resistance by suppressing cell viability and promoting apoptosis.	(170)
	Glioblastoma	MSCs	Anti-miR-9	Multidrug resistance transporters	Reduced drug resistance and enhanced sensitivity to temozolomide (TMZ).	(171)
	Hepatocellular Carcinoma	Bone Marrow MSCs	Grp78 siRNA	Sorafenib resistance pathway	Reversed resistance to sorafenib by targeting Grp78.	(172)
Targeted Therapy Resistance	Chronic Myeloid Leukemia	Bone Marrow MSCs	miR-15a	Bcl-2 and Caspase 3	Promoted resistance to imatinib by reducing apoptosis and enhancing survival pathways.	(173)
	Chronic Lymphocytic Leukemia	Bone Marrow MSCs	EGR1/2/3, MYC	Various pathways	Enhanced resistance to targeted drugs, including ibrutinib and idelalisib.	(174)
Immunotherapy Resistance	Breast Cancer	Bone Marrow MSCs	TGF-β, Semaphorins	PD-L1 overexpression	Promoted immunosuppression by enhancing M2 macrophage polarization and reducing T-cell activation.	(175)
Endocrinotherapy Resistance	Breast Cancer	Adipose - Derived MSCs	miR- 221/222	p27 and ERα signaling	Contributed to tamoxifen resistance by regulating apoptosis and hormone receptor pathways.	(176)
	Prostate Cancer	Umbilic al Cord MSCs	miR-375 siRNA	PTPN4/STAT 3 signaling	Reduced resistance to enzalutamide by inhibiting cell proliferation and migration.	(177)

### 6.6 MSC-EVs as drug-delivery tools for anti-cancer drugs

EVs offer several advantages as drug delivery systems due to their ability to carry diverse bioactive molecules, such as nucleic acids, proteins, and other therapeutic compounds. Their natural biocompatibility, low immunogenicity, and capacity to bypass biological barriers make them highly suitable for targeted drug delivery applications. Compared to traditional drug delivery methods, MSC-derived EVs provide unique advantages, including improved cellular uptake by target cells, extended circulation time, and enhanced stability and protection of therapeutic cargo. Moreover, the specific properties of MSCs, such as their immunomodulatory and tissue repair capabilities, are reflected in the composition and functionality of their EVs. These attributes not only enhance the therapeutic potential of MSC-derived EVs as drug carriers but also contribute to broader therapeutic effects, such as modulating immune responses, promoting angiogenesis, and facilitating tissue regeneration (178, 179).

MSC-derived EVs have a promising future as drug delivery vehicles, but there are still some obstacles to overcome before they can be successfully applied in clinical settings. Key hurdles include improving targeting specificity, ensuring consistent and scalable production, and refining isolation and purification techniques to maximize therapeutic efficacy. Addressing these obstacles is essential to fully realize the potential of MSC-derived EVs in clinical practice (180).

Extensive studies highlight the potential of miRNAs as therapeutic agents in cancer, emphasizing their dual inhibitory and restorative roles. Dysregulated miRNAs, resulting from cancer progression, play critical roles in tumor initiation and development, functioning either as onco-miRs or tumor-suppressor miRNAs (TS-miRNAs). Restoring regulatory miRNAs in cancer cells has shown promising therapeutic effects, such as inhibiting proliferation, migration, invasion, angiogenesis, and metastasis while enhancing chemosensitivity and inducing apoptosis via target gene regulation (19). The success of miRNA-based cancer therapy largely depends on the efficacy of the delivery system. Notably, MSC-derived EVs have been validated as effective carriers for therapeutic miRNAs across various malignancies, presenting a novel, highly promising approach to cancer treatment. However, despite the anticancer potential of MSCs, their role in tumor progression remains controversial and warrants consideration. EVs -based therapies offer a safer alternative to live cell injections, addressing safety concerns. However, advancing the clinical application of MSC-derived EVs requires further research to understand MSC biology, clarify tumor-site homing mechanisms, develop efficacy and safety standards, and refine methods for delivering therapeutic molecules like miRNAs (19). **Table 5** provides a concise overview of the miRNAs used for restoration therapy in various cancers.

**Table 5:** miRNA restoration therapy with MSC-derived exosomes in different cancers.

Cancer type	miRNA	Target genes	Therapeutic effects	Ref.
Breast Cancer	miR-379	COX-2	Reduced tumor size	(181)
	miR-381	LRH-1, Cx43, Sox4, LRP6	Inhibited proliferation, migration, invasion, induced apoptosis	(182)
	miR-34a	Bcl2, c-MET	Decreased proliferation, migration, invasion	(183)
	miR-142	APC, P2X7R	Induced apoptosis, reduced tumor size	(184)
	miR-142-3p	–	Induced apoptosis, inhibited colony formation and tumorigenicity	(185)
	miR-148a	TRIM59	Reduced proliferation, invasion, migration, stimulated apoptosis, inhibited tumor formation and EMT	(186)
	miR-133b	SIRT1, EMP2, MMP14, SOX9	Suppressed proliferation, invasion, migration, inhibited tumor growth	(187)
	miR-584	CYP2J2	Reduced tumor growth	(188)
	miR-146b	MMP16, EGFR, TRAF6	Reduced tumor growth	(189)
Glioma	miR-124a	FOXA2, SCP-1, SOX2	Reduced cell viability, colony formation, migration, improved survival rates, chemosensitivity	(190, 191)
	miR-29a	ROBO1	Inhibited migration, vasculogenic mimicry formation, prolonged survival	(192)
	miR-199a	AGAP2	Inhibited proliferation, invasion, migration, improved chemosensitivity	(193)
	miR-34a	MYCN	Inhibited cell progression, tumorigenesis, induced chemosensitivity	(194)
	miR-9	SHH/PTCH1/MDR1	Reversed chemoresistance, induced cell death	(171)
	miR-4461	COPB2	Reduced migration and invasion	(195)
Colorectal Cancer	miR-16	ITGA2	Suppressed proliferation, migration, invasion, increased apoptosis	(196)
	miR-3940	ITGA6	Inhibited EMT, invasion, tumor growth, metastasis	(197)
	miR-199a	mTOR	Increased chemosensitivity to doxorubicin	(198)
Hepatocellular Carcinoma	miR-122	CCNG1, IGF1R, ADAM10	Induced chemosensitivity to 5-FU and sorafenib	(199)
	miR-451	ADAM10	Inhibited resistance to paclitaxel, progression, EMT, induced apoptosis	(200)
	miR-145	ANGPT2, NEDD9	Inhibited proliferation, invasion, induced apoptosis, reduced tumor size and weight	(201)
Pancreatic Cancer	miR-126	ADAM9	Repressed proliferation, invasion, metastasis, stimulated apoptosis	(202)
	miR-1231	EGFR	Inhibited cell progression, cellular adhesion, tumor growth	(203)
	miR-101	COL10A1	Inhibited cancer progression, reduced tumor growth	(204)
Oral Cancer	miR-185	Akt	Reduced dysplasia, activated apoptotic pathway	(205)
	miR-375	ENAH	Inhibited cancer progression, tumor formation, induced apoptosis, reduced tumor growth	(206)
Esophageal Cancer	miR-139, miR-9	PRC1, ESM1	Repressed tumorigenic characteristics, reduced tumor growth and metastasis (bladder cancer)	(207, 208)

### 6.7 Challenges and future directions

Despite extensive global efforts in academic and pharmaceutical research, current anticancer therapies are effective in treating only a limited number of neoplasms. Terms such as big killers, which refer to tumors with persistently high mortality rates, along with concepts like undruggable cancer targets and chemoresistance, underscore the significant challenges faced in cancer treatment. Moreover, critical factors such as metastasis, tumor microenvironment, tumor heterogeneity, metabolic reprogramming, and resistance to immunotherapy play central roles in modulating tumor responses to therapy. However, these factors continue to lack robust therapeutic interventions or modulators (209).

MSCs are widely used for treating various diseases due to their ability to target damaged tissues, differentiate into multiple cell types, and offer diverse therapeutic effects. However, their use in cancer therapy has shown mixed results, with both anti-tumor and pro-tumor effects observed in preclinical studies. Despite these challenges, recent MSC-based therapies offer hope for personalized, effective cancer treatments. One key advancement is the use of MSCs as “Trojan horses” to deliver therapeutic agents directly to tumors. Understanding the interaction between MSCs and cancer cells is critical for improving the safety of these therapies. Additionally, MSCderived EVs present a promising alternative to live-cell therapy, potentially addressing safety concerns (210, 211). EVs, secretomes, and exosomes are key mediators of cell-to-cell communication, helping maintain physiological balance and influencing disease development. Due to their low immunogenicity, biodegradability, minimal toxicity, and ability to transport bioactive molecules across biological barriers, they are seen as promising alternatives to stem cell therapy. MSC-derived EVs, exosomes, and secretomes have demonstrated regenerative, antiinflammatory, and immunomodulatory properties in treating various human diseases (212).

A comprehensive understanding of the biological roles of MSC-derived extracellular vesicles (MSC-EVs) could pave the way for more effective strategies to overcome or reverse cancer therapy resistance, thereby enhancing treatment outcomes. Additionally, MSC-EVs have emerged as promising carriers for drug and biomolecular delivery, with ongoing research exploring their potential to mitigate the adverse effects of cancer therapies (165).

Several challenges must be addressed before MSC-EVs can be effectively utilized in experimental and clinical settings. First, the complexity and heterogeneity of MSC-EVs necessitate the development of standardized protocols for their nomenclature, classification, isolation, and characterization. Second, safety concerns pose significant barriers to their clinical application, as MSC-EVs have demonstrated both tumor-promoting and tumor-suppressing properties depending on the cancer type and MSC source (158). Studies have reported that MSC-EVs may stimulate cancer cell proliferation, inhibit apoptosis, and promote angiogenesis through the transfer of miRNAs, limiting their clinical potential (213). Third, improving the bioavailability of MSC-EVs is critical, as their tumor-homing properties are compromised by off-target uptake and rapid clearance by macrophages in the mononuclear phagocyte system (MPS), leading to accumulation in organs like the liver, spleen, and lungs. Lastly, the low yield and limited efficiency of natural MSC-EVs in drug delivery hinder their large-scale application (214). While MSCs from sources such as bone marrow, umbilical cord, and adipose tissue are commonly used for EVs production, the difficulty in obtaining adequate samples further restricts their availability for clinical use.

To enhance the therapeutic efficacy of RIPs and MSC-derived EVs in cancer treatment and address their limitations, combining RIPs with MSC-derived EVs offers a promising strategy. MSC-derived EVs, known for their regenerative, anti-inflammatory, and immunomodulatory properties, provide a biologically compatible and low-toxicity delivery system that can improve the bioavailability and targeted delivery of RIPs to tumor cells. The use of EVs can improve the immunogenicity and short half-life of RIPs, while their ability to traverse biological barriers ensures more effective delivery to tumor sites. Previous studies have highlighted the potential synergistic effects of combining EVs with RIPs. Large bioactive molecules, such as dextran and the RIP (saporin), have been loaded into HeLa cell exosome (215, 216).

As a result, the combination of RIPs with MSC-derived EVs represents a novel and innovative method for cancer treatment, with the potential to maximize the strengths of both techniques. This approach may increase therapeutic efficiency, reduce off-target toxicity, and offer a more personalized and effective cancer treatment. Further investigation into this combination therapy is warranted, and it could pave the way for a new class of cancer treatments that harness the benefits of both RIPs and MSC-derived EVs. The combination of MSC-derived EVs and RIPs could enhance the therapeutic efficacy against cancer. MSC-derived EVs can serve as delivery vehicles for RIPs, thereby concentrating the therapeutic effects at the tumor site while minimizing systemic toxicity.

For example, in 2021, the research team developed a novel strategy for intracellular delivery using macropinocytosis-inducible EVs modified with antimicrobial protein CAP18-derived cellpenetrating peptides. They identified that dimerized (sC18)2 peptides, derived from the CAP18 antimicrobial protein, can be easily incorporated into EV membranes and significantly enhance their cellular uptake. By inducing macropinocytosis via glycosaminoglycan-dependent mechanisms, these modified EVs exhibited increased internalization into targeted cells. The technique demonstrated high efficacy in delivering the cytotoxic protein saporin, encapsulated in EVs by electroporation, highlighting its potential for targeted intracellular delivery of biofunctional molecules and advancing EV-based drug delivery systems (217).

The Nakase Research Group also developed saporin-encapsulated exosomes designed for specific receptor targeting and enhanced cytosolic delivery. This was achieved by modifying functional peptides on exosomal membranes. They introduced innovative techniques for exosomebased saporin delivery, providing a detailed explanation of exosomal properties and peptide-based methods (215, 218, 219).

Zuppone et al. (2024) developed a new EV loading protocol that enhances the encapsulation efficiency of therapeutic toxins while preserving EV properties. Unlike conventional methods, their approach utilizes temporary pH alteration with alkaline sodium carbonate, achieving superior cargo incorporation without altering vesicle size, morphology, or uptake. This method outperformed electroporation, particularly for encapsulating the ribosome-inactivating toxin saporin, which remained protected from degradation and exhibited enhanced cytotoxicity against cancer cells. Their findings demonstrate a promising EV-based drug delivery strategy that maintains vesicle integrity while improving therapeutic efficacy (220).

## 7 Rationale for combination therapy in cancer treatment

Advancements in our understanding of cancer biology, including inter- and intra-tumoral heterogeneity and the intricate interactions between tumors and their microenvironment, have highlighted the growing significance of combination therapies in targeting multiple pathways concurrently. Cancer is characterized by a wide range of genetic, epigenetic, proteomic, and metabolomic alterations, which collectively contribute to the diverse outcomes associated with the disease. This diversity often involves the dysregulation of several signaling pathways, even within a single tumor (221). In addition to tumor-specific factors, the dynamic TME plays a crucial role in cancer progression. The TME comprises various cellular and non-cellular components connected through complex pathways that mediate communication among cancer cells, CSCs, and their surroundings. Consequently, a multi-target approach may offer a more effective cancer treatment strategy compared to the traditional “silver bullet” model of targeting a single pathway (222). The rationale for combination therapy stems from key hallmarks of oncogenesis, including the polygenic mutational basis of most malignancies, as well as challenges such as tumor recurrence, metastasis, and the development of resistance to single-agent therapies, including targeted treatments. Monotherapy approaches aimed at specific signaling pathways have demonstrated limited success in addressing these issues. This underscores the urgent need to develop innovative combinatorial strategies to replace or enhance conventional treatment regimens (7).

In the era of -omics technologies and big data, computational methods are emerging as increasingly favorable therapeutic tools. Through signature matching screens, cancer cells’ proteomic, metabolomic, and genomic profiles can be compared with those of treated cells. This allows researchers to predict which treatments are most effective by identifying those that reverse dysregulation and restore a normal -omics profile (7). The widespread adoption of -omics approaches, combined with advancements in data storage, machine learning algorithms, and computational modeling, has significantly enhanced our understanding of cancer biology and the mechanisms of drug action. These innovations enable the generation of disease-related and drugrelated datasets that facilitate the development of computational drug networks. These networks, using in-silico methods, can efficiently predict the efficacy of existing therapies against specific cancer targets and aid in identifying optimal patient responders and relevant disease biomarkers (209). Artificial intelligence (AI), machine learning (ML), and deep learning (DL) also play critical roles in discovering potential therapeutic strategies. For instance, text mining techniques can uncover novel associations between drugs and diseases. A recent study employed text mining of PubMed data to identify metastasis-related genes in cancer and suggest repurposed drugs that may effectively target these genes (223).

Multimodal therapy incorporates combinations of treatment methods to address the multifaceted nature of complex diseases such as cancer. This approach recognizes that a single treatment modality may not adequately tackle all aspects of a disease. By leveraging complementary mechanisms of action, multimodal therapies aim to enhance treatment efficacy, overcome resistance mechanisms, and improve patient outcomes. This strategy is particularly valuable in targeting both cancer cells and their microenvironment, offering a more comprehensive approach to cancer management (7, 224).

## 8 Challenges of combination therapy

The polygenic mutational basis of cancer often renders single-agent therapies inadequate in controlling tumor growth and preventing recurrence, largely due to the development of resistance mechanisms. Consequently, combination therapies have become a preferred strategy (225).

Regulatory approvals for combination treatments typically rely on randomized phase II or III clinical trials demonstrating improved survival outcomes compared to the standard of care. However, designing and implementing combination therapy trials is inherently more complex than monotherapy trials. These trials must carefully account for the intricacies of drug interactions, efficacy, and overall therapeutic benefit (226). The substantial financial, resources, and time demands of clinical trials present a significant barrier to the progression of therapies into phase III studies, particularly when single-agent efficacy is a prerequisite for further evaluation. Singleagent activity is often assessed in randomized trials rather than uncontrolled phase II studies, which are more common in cancer. However, randomized trials require large sample sizes to detect small effects, imposing additional financial burdens (227, 228). This challenge is exacerbated by low patient enrollment rates in clinical trials, with approximately 40% of cancer trials failing due to insufficient patient accrual (229, 230). These factors highlight the inefficiencies in the current clinical trial framework for evaluating combination therapies (227). Importantly, drugs that show limited efficacy as single agents in phase II trials may still exhibit significant therapeutic potential when used in combination. Consequently, these agents are often prematurely excluded from further evaluation, underscoring the need for revised strategies in clinical trial design to better accommodate and assess combination therapies (231).

## 9 Conclusion

Combining RIPs with MSCs/MSC- derived EVs offers a novel approach to cancer therapy by maximizing the benefits of both therapeutic modalities. By targeting tumor cells more precisely, reducing toxicity, and enhancing the pharmacokinetics of RIPs, this method presents a promising avenue for the future of cancer treatment. Further investigation into this combination therapy is warranted, and it could pave the way for a new class of cancer treatments that harness the benefits of both RIPs and MSC- EVs. The combination of MSC- EVs and RIPs could enhance the therapeutic efficacy against cancer. Despite their potential, there are several challenges and limitations associated with using RIPs and MSC- EVs in cancer treatment. One challenge is the need for further research and clinical trials to establish the optimal dosing, administration route, and treatment protocols for these therapies. Additionally, the cost of producing and purifying RIPs can be a limiting factor for their widespread use. For MSCs/MSC-EVs, there are challenges related to their isolation, expansion, and quality control, as well as concerns about their potential to promote tumor growth. These challenges need to be addressed in order to fully realize the potential of RIPs and MSCs/MSC-EVs in cancer treatment. As combination therapies become more central to cancer treatment strategies, the MSCs/MSC- EVs -RIP approach could serve as a pivotal advancement in the fight against cancer. However, further research and rigorous validation are essential to fully understand the mechanisms involved and to optimize the clinical application of this combined strategy.
